# An eco-physiological model of forest photosynthesis and transpiration under combined nitrogen and water limitation

**DOI:** 10.1093/treephys/tpae168

**Published:** 2025-01-10

**Authors:** Peter Fransson, Hyungwoo Lim, Peng Zhao, Pantana Tor-ngern, Matthias Peichl, Hjalmar Laudon, Nils Henriksson, Torgny Näsholm, Oskar Franklin

**Affiliations:** Department of Forest Ecology and Management, Swedish University of Agricultural Sciences, Skogsmarksgränd 17, SE-901 83 Umeå, Sweden; Interdisciplinary Center for Scientific Computing, Heidelberg University, Im Neuenheimer Feld 205, 69120 Heidelberg, Germany; Department of Forest Ecology and Management, Swedish University of Agricultural Sciences, Skogsmarksgränd 17, SE-901 83 Umeå, Sweden; Biodiversity and Natural Resources Program, International Institute for Applied Systems Analysis, Schlossplatz 1, Laxenburg, A-2361, Austria; Department of Forest Ecology and Management, Swedish University of Agricultural Sciences, Skogsmarksgränd 17, SE-901 83 Umeå, Sweden; Department of Environmental Science, Faculty of Science, Chulalongkorn University, 254 Payathai Rd, Wang Mai, Pathumwan, Bangkok 10330, Thailand; Water Science and Technology for Sustainable Environment Research Unit, Chulalongkorn University, Bangkok 10330, Thailand; Department of Forest Ecology and Management, Swedish University of Agricultural Sciences, Skogsmarksgränd 17, SE-901 83 Umeå, Sweden; Department of Forest Ecology and Management, Swedish University of Agricultural Sciences, Skogsmarksgränd 17, SE-901 83 Umeå, Sweden; Department of Forest Ecology and Management, Swedish University of Agricultural Sciences, Skogsmarksgränd 17, SE-901 83 Umeå, Sweden; Department of Forest Ecology and Management, Swedish University of Agricultural Sciences, Skogsmarksgränd 17, SE-901 83 Umeå, Sweden; Department of Forest Ecology and Management, Swedish University of Agricultural Sciences, Skogsmarksgränd 17, SE-901 83 Umeå, Sweden; Biodiversity and Natural Resources Program, International Institute for Applied Systems Analysis, Schlossplatz 1, Laxenburg, A-2361, Austria

**Keywords:** nitrogen uptake, optimality theory, plant hydraulics, Scots pine, stomatal model

## Abstract

Although the separate effects of water and nitrogen (N) limitations on forest growth are well known, the question of how to predict their combined effects remains a challenge for modeling of climate change impacts on forests. Here, we address this challenge by developing a new eco-physiological model that accounts for plasticity in stomatal conductance and leaf N concentration. Based on optimality principle, our model determines stomatal conductance and leaf N concentration by balancing carbon uptake maximization, hydraulic risk and cost of maintaining photosynthetic capacity. We demonstrate the accuracy of the model predictions by comparing them against gross primary production estimates from eddy covariance flux measurements and sap-flow measurement scaled canopy transpiration in a long-term fertilized and an unfertilized Scots pine (*Pinus sylvestris* L.) forest in northern Sweden. The model also explains the response to N fertilization as a consequence of (i) reduced carbon cost of N uptake and (ii) increased leaf area per hydraulic conductance. The results suggest that leaves optimally coordinate N concentration and stomatal conductance both on short (weekly) time scales in response to weather conditions and on longer time scales in response to soil water and N availabilities.

## Introduction

Human-made increases in atmospheric carbon dioxide (CO_2_) concentration have led to rising temperatures and more drought events (IPCC 2014), which have major impacts on gross primary production ($\mathrm{GPP}$) and forest growth. On one hand, one might expect higher temperatures to positively affect biomechanical processes of photosynthesis (Sage and Kubien 2007) and hence growth. On the other hand, more drought events increase the risk of hydraulic failure and higher mortality (McDowell et al. 2008, Ryan 2011). In addition, tree growth is limited by other factors, such as nitrogen (N) availability, which is particularly important in boreal forests in the northern latitudes (Tamm 1991, Binkley and Högberg 2016, Högberg et al. 2017). Thus, interactive effects of water and temperatures on photosynthesis and growth are further influenced by plant nutrition and soil N accessibility, but it is not yet clear how to best incorporate them in process-based models.

Process-based physiological models are well-suited for assessing the response of photosynthesis to different climate drivers. One such model is the well-established Farquhar and von Caemmerer model of leaf photosynthesis (Farquhar et al. 1980, Farquhar and von Caemmerer 1982). To account for resource limitation of photosynthesis, this model can be complemented with models of stomatal conductance and photosynthetic capacity, which is linked to leaf N concentration. Several semi-empirical models have been proposed to model the response of stomata, such as the Ball and Berry model (Ball et al. 1987), where the stomatal conductance (${g}_s$) is linearly related to the quantity $A{H}_r/{C}_a$. Here, $A$ is the carbon assimilation rate, ${H}_r$ is the relative humidity, and ${C}_a$ is the ambient CO_2_ concentration at the leaf surface.

A limitation of the empirical models is that they can be safely applied only within the range of environmental conditions and observations for which they were developed and, thus, may not be accurate under novel conditions or climate change. To overcome this limitation, adaptive models based on optimization principles have been developed. These models assume that the responses of ${g}_s$ and other plant variables to environmental variations are regulated by an optimal trade-off between carbon gain and cost. In the case of ${g}_s$, the apparent cost is the loss of water through transpiration. Based on this premise, Cowan and Farquhar (1977) proposed the optimal water-use efficiency hypothesis where the carbon gain was $A$ and the cost was assumed proportional to leaf transpiration ($E$), i.e., net gain is $A-E/\lambda$, where $\lambda$ is a constant. However, observations have shown that the cost does not merely increase linearly with respect to $E$, but the slope steepens with rising $E$, as a result of increased absolute water potential and thus bringing the vascular system closer to xylem cavitation (Wolf et al. 2016). Mathematically, this means that the derivative of the cost function, with respect to $E$, should be an increasing function of $E$, defined, for example, as a concave-up parabola (Wolf et al. 2016, Anderegg et al. 2018) or a sigmoid (Sperry et al. 2017). Following the approach of Sperry et al. (2017), Eller et al. (2018) proposed the SOX model in which the costs are a function of root–canopy hydraulic conductance, ${k}_{rc}$. The underlying assumption is that the cost will increase as the ${k}_{rc}$ decreases due to the increase in absolute water potential necessary to maintain $E$. A similar assumption is also applied in the model by Sabot et al. (2022*a*).

While the above-mentioned models have only considered the cost associated with water transport, other models have also incorporated the cost of maintaining photosynthetic capacity into the cost term (Friend 1991). Following similar ideas, Prentice et al. (2014) proposed that the cost of maintaining photosynthetic capacity is proportional to ${V}_{c,\max }/A$, where ${V}_{c,\max }$ is the maximum rate of RuBP carboxylation. The drawback of the Prentice et al. (2014) model is similar to that of the Cowan and Farquhar (1977) approach in that the cost associated with transpiration is proportional to $E/A$, thus its slope is not an increasing function of $E$ (Sabot et al. 2022*b*). In contrast, a more recent approach by Joshi et al. (2022) and Flo et al. (2023), called the P-hydro model, optimizes both photosynthetic capacities and has a transpiration cost increasing with $E$ linked to increasing negative plant water potential assumed to cause hydraulic limitation and damage (Joshi et al. 2022). Also, the recent model by Sabot et al. (2022*a*) optimizes not only the trade-off between hydraulic function and photosynthesis but also optimizes photosynthetic N and its distribution between different components, even accounting for the limited adjustment rates and associated delay of leaf N adjustments over time. However, none of the above-mentioned models explicitly accounts for the effects of varying soil N availability.

In this study, we present a new leaf optimization model which combines the cost of maintaining photosynthetic capacity, inspired by the P-hydro model (Joshi et al. 2022), with the hydraulic cost representation of the SOX model. Thus, we optimize not only stomatal conductance as in the SOX model but also the leaf N content. Our model also accounts for the difference in time scale between the regulation of stomatal conductance and leaf N content. This leaf-based optimality model is upscaled to allow calculations of canopy $\mathrm{GPP}$ and transpiration (${E}_c$). In contrast to existing models of this type (Joshi et al. 2022, Sabot et al. 2022*a*), we include the cost of N uptake in order to account for variation in soil N availability. We test and validate the model against observed $\mathrm{GPP}$ from eddy covariance flux measurements and ${E}_c$ estimates from stem sap-flow measurement for a Scots pine (*Pinus sylvestris* L.) forest in northern Sweden, where 13 years of controlled annual fertilization has been administered alongside an untreated reference stand. This setting allows us to test our model with varying soil N availability and variable climate over several years. We show that the model predicts the seasonal pattern of $\mathrm{GPP}$ and ${E}_c$ well. It also predicts the differences between control and N-fertilized stand as a consequence of different carbon costs of N uptake and leaf area per sapwood area.

## Theory and model

### Model description

**Figure 1:**
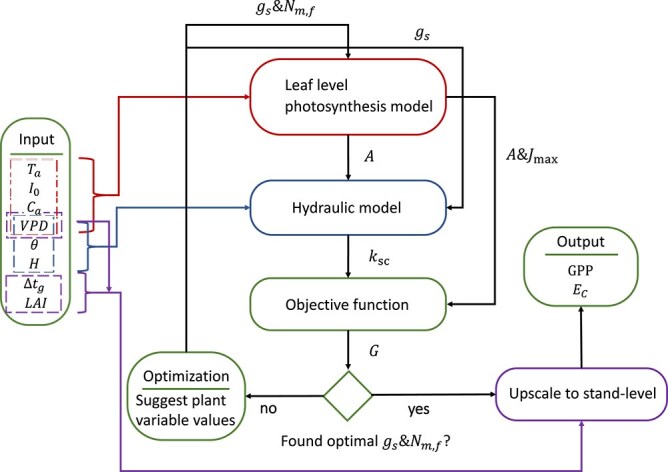
The model is composed of four main modules: leaf level photosynthesis model (LPM), hydraulic model (HM), objective function (OF) and upscaling module (UM). These modules are accompanied by an optimization routine (OR). To run the model, the following inputs are required: ambient air temperature (${T}_a$), above canopy photosynthetic active radiance (${I}_0$), ambient CO_2_ partial pressure (${C}_a$), vapor pressure deficit ($\mathrm{VPD}$), soil volumetric water content ($\theta$), daylight hours ($\Delta{t}_g$), canopy height ($H$) and stand leaf area index ($\mathrm{LAI}$). OR is used to determine the value of the plant variables, stomatal conductance (${g}_s$) and mass-based foliage nitrogen concentration (${N}_{m,f}$) of a leaf situated at the canopy top, by maximizing the trait performance measure ($G$). $G$ is determined by OF with soil-canopy conductance (${k}_{\mathrm{sc}}$), carbon assimilation ($A$) and potential rate of electron transport (${J}_{\mathrm{max}}$) as input. $A$ and ${J}_{\mathrm{max}}$ are calculated by LPM with ${T}_a$, ${I}_0$, ${C}_a$, $\mathrm{VPD}$, ${g}_s$, ${N}_{m,f}$ as inputs. ${k}_{\mathrm{sc}}$ is calculated by HM with $\mathrm{VPD}$, $\theta$, $H$ and ${g}_s$ as inputs. Once an optimum is found, UM upscales leaf-level values to stand-level and we get the model output: per ground area canopy $GPP$, and canopy transpiration (${E}_C$).

A flowchart of the model is provided in Figure 1 and detailed descriptions of the sub-models are presented in the succeeding subsections. Our model calculates daily stand-level canopy GPP and canopy transpiration $\left({E}_c\right)$ based on leaf area index ($\mathrm{LAI}$), canopy height ($H$), and climate data (see Table 1 for a full list of necessary inputs). We assume that physiological response is controlled by two plastic variables: the stomatal conductance (${g}_s$) and foliage N mass-based concentration (${N}_{m,f}$) of a leaf/needle situated at the top of the canopy. The plastic variables are determined by optimization with respect to a fitness proxy, which represents the net carbon gain per leaf area. Central to the optimization is the instantaneous fitness proxy, $G$, which is calculated as the instantaneous leaf-level carbon assimilation of a leaf/needle situated at the top of the canopy ($A$) subtracted by the cost of maintaining transpiration and photosynthetic capacity (the potential electron transportation of a leaf situated at the top canopy, ${J}_{\mathrm{max}}$). The cost of maintaining transpiration reflects drought-related loss of soil-canopy conductance (${k}_{\mathrm{sc}}$) due to xylem embolism, which corresponds to a proportional loss of function of the supported leaf area and its carbon assimilation. The cost of maintaining potential photosynthetic capacity ($N_{r} + N_{u}$) is related to two contributing processes: (i) respiration of a leaf and its supporting roots and stem tissues ($N_{r}$), which is assumed proportional to leaf N content, and (ii) the carbon cost of N uptake ($N_{u}$), which depends on soil N availability. The calculation procedure of $G$ is as follows: first, $A$ and ${J}_{\mathrm{max}}$ are calculated using a mechanistic physiological model. $A$ and ${J}_{\mathrm{max}}$ are functions of climate variables (above canopy photosynthetic active radiation, ${I}_0$, ambient air temperature, ${T}_a$, ambient CO_2_ partial pressure ${C}_a$, vapor pressure deficit $\mathrm{VPD}$), and the two plastic variables. Next, ${k}_{\mathrm{sc}}$ is calculated by the hydraulic model (HM) with $\mathrm{VPD}$, soil volumetric water content ($\theta$), $H$, and ${g}_s$ as input. Using these calculations in an iterative optimization algorithm, the cumulative fitness, i.e. the integral of $G$, over a week is maximized by optimizing ${N}_{m,f}$ and daily ${g}_s$ values (two ${g}_s$ values for each day and one ${N}_{m,f}$ value for the entire week). Subsequently, daily $\mathrm{GPP}$ and ${E}_c$ are calculated by upscaling the leaf-level values to the stand-level. Here, the stand $\mathrm{LAI}$ and the daylight hours ($\Delta{t}_g$) are used as additional input.

**Table 1:** Model input. Value ranges are taken from weather data and modeled size data for the fertilized and control stand at Rosinedal experimental site. The data measurements are from 2015 to 2018, during the growth period.

Symbol	Description	Range (units)
Climate and environmental input		
${T}_a$	Ambient air temperature	−5.1 to 30.8 (°C)
${I}_0$	Above canopy photosynthetic active radiance	1.8–63.5 (mol m^−2^ day^−1^). These values are estimated from solar radiation data using the conversion rate 1000 W m^−2^ ≈ 2300 μmol m^−2^ s^−1^ (2.2.1.a, Landsberg and Sands 2010)
${C}_a$	Ambient CO_2_ partial pressure	38.5–40.9 (Pa)
$\mathrm{VPD}$	Vapor pressure deficit	45.5–1420 (Pa). Estimated from vapor pressure data and calculated saturated vapor pressure values. Saturated vapor pressure is calculated as a function of ${T}_a$ (Alduchov and Eskridge 1996)
$\theta$	Soil volumetric water content	9.9–29.8 (%) (−0.42 to −0.13 MPa soil water potential) for the fertilized stand and 6.7–21.1 (%) (−0.64 to −0.19 MPa soil water potential) for the control stand
$\Delta{t}_g$	Daylight hours	10.2–20.3 (h). The daylight hours are calculated using Eq. (17) from Jenkins (2013)
Stand and tree size input		
$H$	Canopy height	19.07–19.87 (m) for the fertilized stand and 20.86–21.36 (m) for the control stand
$\mathrm{LAI}$	Leaf area index (projected)	2.38–2.45 (m^2^ m^−2^) for the fertilized stand and 2.21–2.30 (m^2^ m^−2^) for the control stand

### Leaf level photosynthesis model

The leaf level carbon assimilation is calculated in a standard fashion as a balance between the rate of assimilation (carbon demand) and the mass transport of CO_2_ into the leaf through stomatal and mesophyll conductance (carbon supply). The assimilation rate $\mathrm{A}$ (mol m^−2^ s^−1^) is calculated as the minimum of electron transport-limited assimilation rate, ${A}_j$, and the carboxylation-limited assimilation rate, ${A}_c$, (Farquhar et al. 1980). We assume co-limitation, i.e. that ${A}_c={A}_j$ (coordination hypothesis, Chen et al. 1993, Maire et al. 2012, Wang et al. 2017, Smith et al. 2019), thus,


(1) \begin{equation*} \mathrm{A}=\frac{J}{4}\frac{c_i-{\varGamma}^{\ast }}{c_i+2{\varGamma}^{\ast }}. \end{equation*}

In Eq. (1), ${c}_i$ (Pa) is the intercellular partial pressure of CO_2_, ${\varGamma}^{\ast }$ (Pa) is the CO_2_ compensation point, and $J$ (mol m^−2^ s^−1^) is the rate of electron transportation; see Eq. (2).


(2) \begin{equation*} J=\frac{\alpha{I}_{\mathrm{I}}+{J}_{\mathrm{max}}-\sqrt{\alpha^2{I_{\mathrm{I}}}^2+2\alpha{I}_{\mathrm{I}}{J}_{\mathrm{max}}\left(1-2{\theta}_J\right)+{J}_{\mathrm{max}}^2}}{2{\theta}_J}. \end{equation*}

In Eq. (2), ${I}_{\mathrm{I}}$ (mol m^−2^ s^−1^) is the irradiance incident on a leaf, ${J}_{\mathrm{max}}$ (mol m^−2^ s^−1^) is the potential electron transportation, ${\theta}_J$ (−) is a measure of the curvature of the light response curve and $\alpha$ (−) is the quantum yield.

The mass transportation of CO_2_ into the chloroplast through stomatal and mesophyll conductance is given by Fick’s law:


(3) \begin{equation*} A=g\frac{\left({c}_{\mathrm{a}}-{c}_{\mathrm{i}}\right)}{P}. \end{equation*}

In Eq. (3), $g$ (mol m^−2^ s^−1^) is the combined stomatal (${g}_s$) and mesophyll (${g}_m$) conductance, and $P$ is the atmospheric pressure (Pa). We assume that ${g}_m$ is proportional to ${g}_s$, thus $g\propto{g}_s$. Specifically, we assume $g\approx 0.42{g}_s$ (Wang et al. 2017). Because the demand and the supply equations need to be balanced, we get:


(4) \begin{align*} \frac{g}{P}\left({c}_{\mathrm{a}}-{c}_{\mathrm{i}}\right)=\frac{J}{4}\frac{c_i-{\varGamma}^{\ast }}{c_i+2{\varGamma}^{\ast }}\iff& {c}_{\mathrm{i}}^2+\left[\frac{JP}{4g}+2{\varGamma}^{\ast }-{c}_{\mathrm{a}}\right]{c}_{\mathrm{i}} \nonumber \\ &-\left[\frac{JP}{4g}{\varGamma}^{\ast }+2{c}_{\mathrm{a}}{\varGamma}^{\ast}\right]=0. \end{align*}

Eq. (4) is a quadratic equation and ${c}_i$ is given by the greater of the two roots. Thus, we have an expression for ${c}_i$ as a function of $g$, ${I}_{\mathrm{I}}$ and ${J}_{\mathrm{max}}$, i.e., ${c}_i\left(g,{I}_{\mathrm{I}},{J}_{\mathrm{max}}\right)$. Similarly, we get an expression for the carbon assimilation (Eq. (5)) by substituting Eq. (4) into Eq. (3). 


(5) \begin{equation*} A\left(g,{I}_{\mathrm{I}},{J}_{\mathrm{max}},{c}_{\mathrm{a}}\right)=\frac{g}{P}\left({c}_{\mathrm{a}}-{c}_{\mathrm{i}}\left(g,{I}_{\mathrm{I}},{J}_{\mathrm{max}}\right)\right). \end{equation*}

#### Temperature dependency of photosynthetic parameters and its acclimation to annual temperature cycle

We use the Arrhenius equation and the model from Tarvainen et al. (2018) to estimate the short-term temperature dependency of the photosynthetic parameters. Specifically, the temperature responds of ${\varGamma}^{\ast }$, is modeled by using the Arrhenius equation (Landsberg and Sands 2010),


(6) \begin{equation*} {\varGamma}^{\ast }={\varGamma}_{\mathrm{ref}}^{\ast }\ \exp \left(\frac{E_{A,\varGamma}\left(T-{T}_{\mathrm{ref}}\right)}{TR{T}_{\mathrm{ref}}}\right). \end{equation*}

In Eq. (6), $R=8.314$ (J K^−1^ mol^−1^) is the gas constant, ${E}_{A,\varGamma }$ (J) is the activation energy of the parameter, $T$ is the temperature (K), and ${\varGamma}_{\mathrm{ref}}^{\ast }$ is the parameter value at a reference temperature ${T}_{\mathrm{ref}}$ (298 K).

We use equation 4 from Tarvainen et al. (2018) to model the short-term temperature responds of ${J}_{\mathrm{max}}$,


(7) \begin{align*} {J}_{\mathrm{max}}=&\ {J}_{\max, \mathrm{opt}}{f}_{\mathrm{Jmax}}(T) \nonumber \\ =&\ {J}_{\max, \mathrm{opt}}\frac{E_{D,J}\exp \left(\frac{E_{A,J}\left(T-{T}_{\mathrm{opt}}\right)}{TR{T}_{\mathrm{opt}}}\right)}{E_{D,J}-{E}_{A,J}\left(1-\exp \left(\frac{E_{D,J}\left(T-{T}_{\mathrm{opt}}\right)}{TR{T}_{\mathrm{opt}}}\right)\right)}. \end{align*}

In Eq. (7), ${E}_{D,J}$ (J) is the deactivation energy, ${E}_{A,J}$ (J) is the activation energy and ${J}_{\mathrm{max,opt}}$ is the value of ${J}_{\mathrm{max}}$ at optimal temperature ${T}_{\mathrm{opt}}$ (K).

On a longer timescale, parameters may acclimate to the annual temperature cycle. Specifically, the magnitude of the light response curve follows the trend of the temperature cycle while the shape of the curve remains constant (Hari and Mäkelä 2003, Mäkelä et al. 2004). Mäkelä et al. (2004) enforced this property by assuming that the quantum yield is proportional to ${J}_{\mathrm{max}}$. For our model, we achieve the same effect by assuming that both ${J}_{\mathrm{max}}$ and the quantum yield, $\alpha$, follow the same seasonal cycle, specifically, $\alpha ={X}_t{\alpha}_{\mathrm{season}}$ and ${J}_{\mathrm{max}}={X}_t{J}_{\max, \mathrm{season}}\left({T}_a,{N}_a\right)$. Here, ${\alpha}_{\mathrm{season}}$ and ${J}_{\max, \mathrm{season}}\left({T}_a,{N}_{m,f}\right)$ are parameters representing the seasonal apex of $\alpha$ and ${J}_{\mathrm{max}}$, respectively, and ${X}_t\in \left[0,1\right]$ is a variable accounting for the reduction of $\alpha$ and ${J}_{\mathrm{max}}$ due to the seasonal variation in temperature. Note that ${J}_{\max, \mathrm{season}}\left({T}_a,{N}_{m,f}\right)$ depends on the ambient air temperature, ${T}_a$, and the N concentration per leaf mass, ${N}_{m,f}$. If these equations are substituted into Eq. (2) we get Eq. (8).


(8) \begin{align*}& J=\ \frac{X_t}{2\theta_{J}}\Bigg[{\alpha}_{\mathrm{season}}{I}_{\mathrm{I}}+{J}_{\max, \mathrm{season}}\left({T}_a,{N}_{m,f}\right)- \nonumber \\ & \sqrt{{\alpha_{\mathrm{season}}}^2{I_{\mathrm{I}}}^2+2{\alpha}_{\mathrm{season}}{J}_{\max, \mathrm{season}}\!\left(\!{T}_a,{N}_{m,f}\!\right){I}_{\mathrm{I}}\!\left(\!1\!-2\theta_J \!\right)+{J}_{\max, \mathrm{season}}{\left(\!{T}_a,{N}_{m,f}\!\right)}^2}\ \Bigg]. \end{align*}

The time-dependent variable ${X}_t$ (−) is a function of the delayed ambient air temperature, ${S}_t$ (°C), see Eq. (9).


(9) \begin{equation*} {X}_t=\left\{\begin{array}{@{}ll@{}}0,& {S}_t\le{S}_{\mathrm{min}},\\{}\frac{S_t-{S}_{\mathrm{min}}}{\Delta S},& {S}_{\mathrm{min}}<{S}_t<{S}_{\mathrm{min}}+\Delta S\\{}1,& {S}_t\ge{S}_{\mathrm{min}}+\Delta S.\end{array}\right., \end{equation*}

In Eq. (9), ${S}_{\mathrm{min}}$is a parameter representing the minimum threshold for the activation of photosynthesis, and $\Delta S\ge 0$ is a parameter controlling when the photosynthetic capacity reaches its seasonal peak. Thus, ${J}_{\mathrm{max}}$ increases linearly with respect to ${S}_t$ in the temperature range ${S}_{\mathrm{min}}<{S}_t<{S}_{\mathrm{min}}+\Delta S.$ The delayed temperature, ${S}_t,$ is the effective temperature to which the photosynthesis has acclimated to, i.e., the temperature that determines the level of activation of photosynthesis (${X}_t$, Eq. (9)). Because this acclimation takes time, ${S}_t$ lags behind the current temperature, which is modeled using a first order delay dynamics model (Mäkelä et al. 2004, 2008):


(10) \begin{equation*} {S}_t=\left(1-\frac{1}{\tau}\right){S}_{t-1}+\frac{1}{\tau }{T}_t,\kern0.5em {S}_0={T}_0. \end{equation*}

In Eq. (10), ${T}_t$ is the ambient air temperature at time $t$, ${T}_0$ is an initial temperature of a temperature time series, and $\tau$ is a parameter controlling the temperature delay; a higher value of $\tau$ equals a longer delay in the temperature response.

#### The effect of N concentration on the photosynthetic capacity

We assume that the seasonal apex of ${J}_{\max, \mathrm{opt}}$ is proportional to the per leaf-mass N concentration, ${N}_{m,f}$, i.e. ${J}_{\max, \mathrm{opt}}\left({N}_{m,f}\right)={a}_{J\max }{N}_{m,f}$ (Franklin 2007, Landsberg and Sands 2010). Here, ${a}_{J\max }$ is a proportionality parameter. Thus, ${J}_{\max, \mathrm{season}}\left({T}_a,{N}_{m,f}\right)={J}_{\max, \mathrm{opt}}\left({N}_{m,f}\right){f}_{\mathrm{Jmax}}\left({T}_a\right)$ and ${J}_{\mathrm{max}}={X}_t{J}_{\max, \mathrm{opt}}\left({N}_{m,f}\right){f}_{\mathrm{Jmax}}\left({T}_a\right)$.

### Hydraulics model

If ${g}_s$ and $\mathrm{VPD}$ are given, we can calculate the canopy transpiration per leaf area, $E$:


(11) \begin{equation*} E=\frac{1.6{g}_s \mathrm{VPD}}{P}. \end{equation*}

In Eq. (11), $P$ is the atmospheric pressure (Pa). We assume that the water flow between root and leaf is in steady-state and negligible non-stomatal water loss, which means that $E$ equals the water uptake. We use Darcy’s law to calculate the canopy water potential, ${\psi}_c$ (MPa), as a function of soil water potential, ${\psi}_s$, and $E$ (Eller et al. 2018):


(12) \begin{equation*} {\psi}_c={\psi}_s- H\rho g 10^{-6}-\frac{E}{k_{\mathrm{sc}}}. \end{equation*}

In Eq. (12), $\rho$ = 997 (kg m^−3^) is the density of water, $g$ = 9.82 (m s^−2^) is the gravitational acceleration and ${k}_{sc}$ (mol m^−2^ leaf s^−1^ MPa^−1^) is the soil-canopy conductance. ${k}_{\mathrm{sc}}$ decreases from a potential maximal value, ${k}_{\mathrm{sc},\max }$, as water potential, $\psi$, declines according to the vulnerability function, $P\left(\psi \right)$:


(13) \begin{equation*} P\left(\psi \right)=\frac{k_{\mathrm{sc}}}{k_{\mathrm{sc},\max }}={\left(\frac{1}{2}\right)}^{{\left(\frac{\psi }{\psi_{50,\mathrm{sc}}}\right)}^{b_{\mathrm{sc}}}}. \end{equation*}

In Eq. (13), ${\psi}_{50,\mathrm{sc}}$ is the water potential resulting in half of the maximum conductivity, i.e. $P\left({\psi}_{50,\mathrm{rc}}\right)=0.5$, and ${b}_{\mathrm{sc}}$ is a shape parameter controlling how fast ${k}_{\mathrm{sc}}$ decreases with the water potential.

We calculate ${\psi}_s$ from the effective soil saturation, ${S}_e$ (−), by applying equation 2.19 from Jansson and Karlberg (2011):


(14) \begin{equation*} {\psi}_s={\psi}_a\ {S}_e^{-1/\lambda }. \end{equation*}

In Eq. (14), ${\psi}_a$ is the air-entry tension and $\lambda$ (−) is the pore size distribution index of the soil.

The effective saturation is a function of soil water content (Jansson and Karlberg 2011), $\theta$ (−):


(15) \begin{equation*} {S}_e=\frac{\theta -{\theta}_r}{\theta_s-{\theta}_r}. \end{equation*}

In Eq. (15), ${\theta}_s$ is the saturated soil water content and ${\theta}_r$ is the residual water content.

We calculate ${k}_{rc}$ by solving Eq. (16) (Sperry and Love 2015).


(16) \begin{equation*} {k}_{\mathrm{sc}}={k}_{\mathrm{sc},\max}\frac{\int_{\psi_c}^{\psi_{c, pd}}P\left(\psi \right) d\psi}{\psi_{c, pd}-{\psi}_c}. \end{equation*}

In Eq. (16), ${\psi}_{c, pd}={\psi}_s- H\rho g 10^{-6}$ is the pre-dawn canopy water potential. We use Simpson’s 1/3 rule to approximate the integral in Eq. (16) and the equation is solved by applying a fix-point iteration method.

### Plant optimization

We define the instantaneous fitness proxy, $G$ (mol m^−2^ s^−1^), as the instantaneous carbon assimilation rate at top of the canopy, $A=A\left({g}_{\mathrm{s}},{I}_{{\mathrm{I}}_0},{J}_{\mathrm{max}}\right)$, times a reduction factor ${k}_{\mathrm{cost}}$, representing the effect of reduced plant conductance under water stress (Eller et al. 2018), minus the cost of maintaining ${J}_{\mathrm{max}}$, i.e. $({N}_r+{N}_u){J}_{\mathrm{max}}$. Thus,


(17) \begin{align*} G=&\ A-A\left(1-{k}_{\mathrm{cost}}\right)-\left({N}_r+{N}_u\right){J}_{\mathrm{max}} \nonumber \\ =&\ A{k}_{\mathrm{cost}}-\left({N}_r+{N}_u\right){J}_{\mathrm{max}}. \end{align*}

In Eq. (17), ${N}_r$ (−) represents the leaf respiration cost as the ratio between dark respiration ($R_d$) and ${J}_{\mathrm{max}}$, which is linked to leaf N because photosynthetic capacity and ${J}_{\mathrm{max}}$ increases with leaf N concentration associated with photosynthetic proteins. ${N}_r$ was estimated based on measured ${J}_{\mathrm{max}}$ and $R_d$ (night and daytime values) in Scots pine (Kellomäki and Wang 1997). Because ${N}_r$ is based on the ratio of fundamental leaf biochemical processes with similar climatic responses (Wang et al. 2020) it is relatively constant among species and climate conditions. ${N}_u$ represents the carbon investment (fine-roots, mycorrhiza, exudation) for nutrient uptake required to construct and maintain ${J}_{\mathrm{max}}$which is expected to strongly depend on soil N availability.

The costs of hydraulic risks and damage are represented by the parameter ${k}_{\mathrm{cost}}=\left({k}_{\mathrm{sc}}-{k}_{\mathrm{crit}}\right)/\left({k}_{\mathrm{sc},\max }-{k}_{\mathrm{crit}}\right)$ (Supplementary material of Eller et al. 2018). Based on the commonly observed lethal loss of conductivity of 88% (Liang et al. 2021), here we assumed ${k}_{\mathrm{crit}}=0.12{k}_{\mathrm{sc},\max }$ and the $A\left(1-{k}_{\mathrm{cost}}\right)$ cost term we assume that: (i) each fraction of ${k}_{\mathrm{sc}}$ corresponds to an equal loss in functional leaf area and thus assimilation loss and (ii) in the event of hydraulic failure and fatal embolism, i.e., when ${k}_{rc}$ decreases and approaches 0, the loss should be equal to the total carbon gain.

While $G$ represents the instantaneous fitness reward, we assume that ${N}_{m,f}$ and ${g}_s$ regulate such that the accumulative fitness, i.e., the integration of $G$ over time, is maximized. Furthermore, we assume that: (i) ${N}_{m,f}$ optimizes on a weekly time scale and ${g}_s$ on a sub-daily time scale. (ii) ${N}_f$ is constant over a week’s period. (iii) The day-to-day change of the weather variables within a week is neglectable compared to the within-day variation. (iv) The daily integration of $G$ can be approximated by the sum of two instantaneous function values according to the two-segment daily model (SDM-2, Wang et al. 2014). The original segmented daily model assumed that the nonlinear response of $A$ can be approximated by a piecewise linear function, i.e. the response curve can be approximated by a number of line segments and that weather variables (specifically radiation, temperature and relative humidity) follow a sine function. With these assumptions, we propose a two-step optimization routine (OR). In the first step, we optimize ${N}_{m,f}$ and two ${g}_s$ values to maximize the integral of $G$ over a specified week (long-term optimization). The two ${g}_s$ values represent the within-day variation of stomatal conductance (one ${g}_s$ value for each segment in SDM-2) for an average day within the specific week. In the second step, we maximize the daily integral of $G$ for each day in the specified week by re-optimizing the two ${g}_s$ value for each day (fine-tuning). Here, we use the optimal weekly ${N}_{m,f}$ value from the previous step as input, and the two average-day ${g}_s$ values from the first step are used as initial guesses for the optimization algorithm. Additional information regarding plant optimization is available in the supplementary information (Methods S1 available as Supplementary data at *Tree Physiology* Online). In order to use the SDM-2 approximation, we need to estimate within-day values for the weather variables. To this end, we use the same trigonometric functions from Wang et al. (2014) to model the diurnal change of the ambient temperature and radiation; the remaining weather variables are either calculated from these ($\mathrm{VPD}$) or are assumed to be constant during the day (${C}_a$ and $\theta$), see Methods S1 for more information*.*

The optimization problem is solved by using the implementation of Broyden–Fletcher–Goldfarb–Shanno (BFGS) algorithm from the *Optim.jl* package (Mogensen and Riseth 2018). We search the optimum in the range $0.007\le{N}_{m,f}\le 0.05$ and $0.001\le{g}_s\le{g}_{s,\mathrm{crit}}$ for each ${g}_s$ and ${N}_{m,f}$ value. Here, ${g}_{s,\mathrm{crit}}$ is the stomatal conductance which results in $P\left({\psi}_c\right)=0.12$.

### Upscaling to stand-level

#### Stand level primary production

We assume that ${J}_{\mathrm{max}}$ and ${g}_s$ acclimate to irradiance, resulting in a proportional relationship with irradiance level (Landsberg and Sands 2010). Then, the instantaneous $\mathrm{GPP}$ per ground area of canopy vegetation ($\mathrm{GPP}$ of trees), $\mathrm{GP}{\mathrm{P}}_c$, is calculated as:


(18) \begin{align*} \mathrm{GP}{\mathrm{P}}_c=&\ {\int}_0^{\mathrm{LAI}}A\left({g}_s,{I}_{\mathrm{I}},{J}_{\mathrm{max}}\right)d\mathrm{LAI} \nonumber \\ =&\frac{1-\exp \left(-k\times \mathrm{LAI}\right)}{k}A\left({g}_{\mathrm{s}},{I}_{{\mathrm{I}}_0},{J}_{\mathrm{max}}\right). \end{align*}

In Eq. (18), $\mathrm{LAI}$ is the leaf area index (projected area), and $k$ and ${I}_{\mathrm{I}}$ are the light extinction coefficient and irradiance incident on a leaf, respectively, see Methods S2 (available as Supplementary data at *Tree Physiology* Online) for further details. Analog to the plant optimization routine, we employ the SDM-2 approximation (Wang et al. 2014) to calculate daily $\mathrm{GPP}$ value from instantaneous values. In order to compare with eddy-covariance data (ecosystem $\mathrm{GPP}$, $\mathrm{GP}{\mathrm{P}}_e$), we accounted for understory vegetation $\mathrm{GPP}$, $\mathrm{GP}{\mathrm{P}}_g$, to calculate $\mathrm{GP}{\mathrm{P}}_e=\mathrm{GP}{\mathrm{P}}_c+\mathrm{GP}{\mathrm{P}}_g$. We assume that light use efficiency ($\mathrm{LUE}$, defined as $\mathrm{GPP}$/absorbed light) is the same for both vegetation layers (Tian et al. 2021). $\mathrm{GP}{\mathrm{P}}_c$ can then be upscaled by using an upscale factor, $\zeta =\mathrm{GP}{\mathrm{P}}_e/\mathrm{GP}{\mathrm{P}}_c$. The value of $\zeta$ depends on the understory vegetation $\mathrm{LAI}$ and corresponding light extinction coefficient (see Methods S3 available as Supplementary data at *Tree Physiology* Online for more information). We estimate that $\zeta \approx 1.2$ for the fertilized stand and $\zeta \approx 1.13$ for the control (see Table S1 available as Supplementary data at *Tree Physiology* Online). These corresponding contribution of $\mathrm{GP}{\mathrm{P}}_g$ to $\mathrm{GP}{\mathrm{P}}_e$ was 17% and 12% for the fertilized stand and control, respectively. This is in line with previous estimates (Chi et al. 2021). Hereafter, we will refer to $\mathrm{GP}{\mathrm{P}}_e$ as simply $\mathrm{GPP}$.

#### Canopy transpiration

We neglect the effects of boundary layer conductance (combined leaf and canopy boundary layer); thus, the instantaneous canopy transpiration ${E}_c$ is calculated as:


(19) \begin{equation*} {E}_c={g}_C\frac{VPD}{P}. \end{equation*}

In Eq. (19), ${g}_C$ is the canopy conductance. The canopy conductance is calculated as:


(20) \begin{equation*} {g}_C=1.6{\int}_{\!\!\!\!0}^{\mathrm{LAI}}{g}_sd\mathrm{LAI}=1.6{g}_{s,\mathrm{top}}\frac{1-\exp \left(-k\times \mathrm{LAI}\right)}{k}. \end{equation*}

See Methods S2 (available as Supplementary data at *Tree Physiology* Online) for further details. Again, we employ the SDM-2 approximation (Wang et al. 2014) to calculate the daily values for ${E}_c$.

### Data

**Figure 2:**
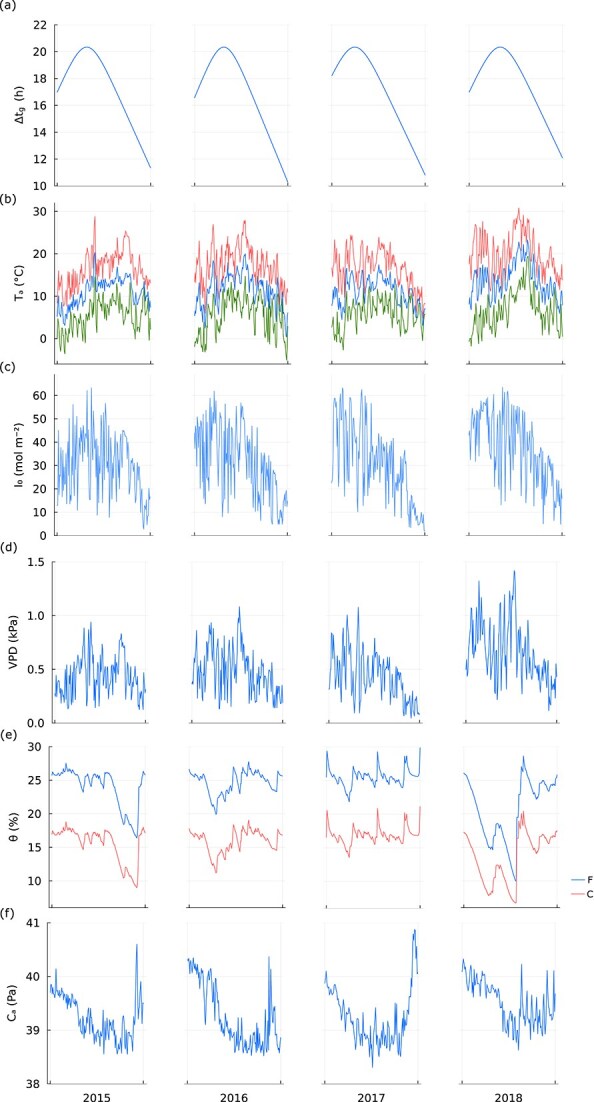
Weather data for the fertilized (F) and control (C) stand at the experimental site Rosinedal during the growth period between 2015 and 2018. Climate drivers: daylight hours ($\Delta{t}_g$) (a), ambient air temperature (${T}_a$) (daily max, red curve, daily min, green curve and daily mean, blue curve) (b), daily photosynthetic active radiance (${I}_0$) (c), vapor pressure deficit ($\mathrm{VPD}$) (d), soil volumetric water content ($\theta$) of the fertilized stand (F) and the control (C) (e), ambient CO_2_ partial pressure (${C}_a$) (f). These values were used as input for our model.

The data used for model calibration are based on measurements from the experimental site Rosinedal (64°10′ N, 19°45′ E). The site is a 90-year-old naturally regenerated Scots pine forest, regenerated with seed trees in 1920–25. In 1955, the stand was pre-commercially thinned, followed by thinnings in 1976 and 1993. The experiment was established in 2005 and annual N fertilization started in 2006 with addition of 100 kg N ha^−1^ year^−1^ from 2006 to 2011, and reduced to 50 kg N ha^−1^ year^−1^ in 2012 (Lim et al. 2015). We used weather data and measured tree dimensions from the fertilized and the control stand as input for the model; see Table 1 for a full list of input variables and Figure 2 for a depiction of the weather data time series. Briefly, stem diameter was measured at 1.3 m (DBH) annually for all trees in each of the three mensuration stands (1000 m^2^) for each treatment stand. Tree height and length of live crown were measured on 20 trees per stand, using Vertex 4 Ultrasonic Hypsometer (Haglöf Inc., Sweden). We developed a relationship between height and DBH following the recommendation of Näslund (1947); parameters of the function were estimated each year for each stand and then applied to all individual trees. Based on destructive tree harvests in June 2006, October 2012 and October 2018, we developed allometric equations for foliage, stem and branch biomass. From 2012 and 2018 harvest samples, subsets of fresh foliage samples were scanned and dried to estimate specific leaf areas. Biomass of each component was predicted based on a combination of the tree dimensions and the developed allometric equations. We estimated leaf area index by multiplying foliage biomass and specific leaf area estimates. Model outputs were calculated using daily data and validated against the eddy-covariance based ecosystem $\mathrm{GPP}$ estimates (Zhao et al. 2022) and ${E}_C$ values for the growth periods of 2015–18. The growing seasons were assumed to start when daily mean temperature was ≥5 °C for five successive days and end when daily mean temperature was <5 °C for five successive days. The ${E}_C$ values are estimated using the empirical model from Tor-ngern et al. (2017) with corresponding parameter estimates for the study site provided in that paper. Daily partial pressure of atmospheric CO_2_ [CO_2_] was collected from Integrated Carbon Observatory System (ICOS) tower at Svartberget, 10 km north of the site (www.icos-sweden.se). We used Level 1 datasets (basic quality control) and Level 2 datasets (the full quality control) at 150 m height. Level 1 was available from 2015 throughout 2020, excepting 2018 data, while Level 2 was available from 2017. We based our [CO_2_] input on the Level 1 data with gap-filling the 2018 missing data using a correlation between Level 1 and Level 2 for overlapped measurement points (2017 and 2020, [CO_2_] at Level 1 = [CO_2_] at Level 2 × 0.783 + 781; *n* = 742 daily mean [CO_2_]).

### Parameter estimation

The set of unknown parameters of the model, $\boldsymbol{\theta}$ (see Table 3 for a full list of estimated parameters) that could not be estimated based on measurements were estimated by fitting the model to observations. The remaining model parameters were taken from other sources, see Table 2. To minimize the effect of data outliers, we assume that model and measurement errors are Laplace distributed (Tian et al. 2021), i.e. ${y}_{i,j,k}-M{\left({X}_{j,k},\boldsymbol{\theta} \right)}_{i,j,k}={e}_{i,j,k}\sim \mathrm{Laplace}\left(0,{a}_{i,j}+{b}_{i,j}M{\left(\boldsymbol{\theta} \right)}_{i,j,k}\right)$. Here, ${y}_{i,j,k}$ denotes the response variables (the measured variables), ${X}_{j,k}$ are the collection of explanatory variables (tree size and climate data). $M$ is the model output, ${e}_{i,j,k}$ are the model errors, $\mathrm{Laplace}\left(\mu, b\right)$ is the Laplace distribution with location parameter $\mu$ and scale parameter $b>0$. The parameters ${a}_{i,j}$ and ${b}_{i,j}$ control the variance of the error distribution and we assume that the scale parameter is linear with respect to the model output (Tian et al. 2021). We also use data type-specific weights, ${w}_i$, to weigh the importance of different data types. The subscripts $i,j,k$ denotes the data type ($\mathrm{GPP}$, ${E}_C$ or ${N}_{m,f}$), the stand treatment (control or fertilized) and the data index (individual observations), respectively. This assumption leads to the following likelihood function:


(21) \begin{align*} L\left(Y\right|\boldsymbol{\theta}, \boldsymbol{a},\boldsymbol{b})=&\ \prod_i\prod_j\prod_k \left[\frac{1}{2\left({a}_{i,j}+{b}_{i,j}M{\left(\boldsymbol{\theta} \right)}_{i,j,k}\right)}\right. \nonumber \\ & \left.\times \exp \left(-\frac{\left|{e}_{i,j,k}\right|}{a_{i,j}+{b}_{i,j}M{\left(\boldsymbol{\theta} \right)}_{i,j,k}}\right)\right]^{w_i}. \end{align*}

**Table 2:** Parameter values used in the model.

Parameter	Description	Value (units)	Reference/note
GPP model parameters			
${\varGamma}_{\mathrm{ref}}^{\ast }/{E}_{A,\varGamma }$	The CO_2_ compensation point at 25 °C/Activation energy of ${\varGamma}^{\ast }$	4.17 (Pa)/23.42 (kJ mol^−1^)	Table 3. 1 in Landsberg and Sands (2010)
${E}_{A,J}$ /${E}_{D,J}$/${T}_{\mathrm{opt}}$	Activation energy of ${J}_{\mathrm{max}}$/deactivation energy of ${J}_{\mathrm{max}}$/optimal temperature for ${J}_{\mathrm{max}}$	47.4 (kJ mol^−1^)/200 (kJ mol^−1^)/305 (K)	(Tarvainen et al. 2018)
${\theta}_J$	Curvature of the light response curve	0.7 (−)	Table 1 in Landsberg and Sands (2010)
$k$	Light extinction coefficient	0.52 (−)	(Tian et al. 2021)
$m$	Leaf transmittance	0.05 (−)	Ch. 5.1.1 in Landsberg and Sands (2010)
Hydraulics model parameters			
${\psi}_{50,\mathrm{sc}}$ /${b}_{\mathrm{sc}}$	Water potential which causes 50% loss in soil-canopy hydraulic conductivity/sensitivity of soil-canopy hydraulic conductivity to water potential	−2.7 (MPa)/2.15 (−)	${\psi}_{50,\mathrm{sc}}$ and ${b}_{\mathrm{sc}}$ are estimated from Aguade et al. (2015)
${\theta}_s$ /${\theta}_r$	Saturated soil water content/residual water content	0.41 (−)/0.006 (−)	From unpublished Pf-curve
${\psi}_a$ /$\lambda$	Air-entry tension/pore size distribution index	−0.098 (MPa)/1 (−)	From unpublished Pf-curve
Nitrogen cost parameter			
${N}_r$	Ratio between dark respiration and ${J}_{\mathrm{max}}$	0.0056 (−)	Estimated from Kellomäki and Wang (1997)
Variance parameters			
${a}_{E_c,\mathrm{j}}$ /${b}_{E_c,j}$	The parameters which control the variance of the error distribution for ${E}_c$ (mm day^−1^/−)	$0.069$ /$0.15$ ($j=\mathrm{Fertilized}$) $0.043$/$0.18$ ($j=\mathrm{Control}$)	Tian et al. 2021
${y}_{N_{m,f,j,k}}$ /${a}_{N_{m,f},j}/{b}_{N_{m,f,j}}$	The response variable and parameters which control the variance of the error distribution for ${N}_{m,f}$ (Kg Kg^−1^/Kg Kg^−1^/−)	$0.021$ /$0.0023$/$0$ ($j=\mathrm{Fertilized}$ and for all $k$) $0.012$/$0.0023$/$0$ ($j=\mathrm{Control}$ and for all $k$)	Estimated from data Lim et al. (2015)

In Eq. (21), ${a},{b}$ are collections of ${a}_{i,j}$ and ${b}_{i,j}$ values, respectively. The values for ${y}_{N_{m,f},j,k}$, ${a}_{N_{m,f},j}$ and ${b}_{N_{m,f},j}$ were estimated from data Lim et al. (2015) and the values of ${a}_{E_c,j}$ and ${b}_{E_c,j}$ were taken from Tian et al. (2021) (Table 2), while ${a}_{\mathrm{GPP},j}$ and ${b}_{\mathrm{GPP},j}$ were estimated in conjunction with the unknown model parameters and we used ${w}_{E_C}={w}_{N_{m,f}}=1.0$ and ${w}_{\mathrm{GPP}}=1.5$.

The parameter estimates, $\left({{\boldsymbol{\theta}}}^{\ast},{a_{\mathrm{GPP},j}}^{\ast },{b_{\mathrm{GPP},j}}^{\ast}\right)$, were determined by maximization of the likelihood function, i.e., ${{\boldsymbol{\theta}}}^{\ast },{a_{\mathrm{GPP},j}}^{\ast },{b_{\mathrm{GPP},j}}^{\ast }=\arg{\ \max }{_{\boldsymbol{\theta}, {a}_{\mathrm{GPP},j},{b}_{\mathrm{GPP},j}}} \ L\left(Y\right|{\boldsymbol{\theta}}, \boldsymbol{a},\boldsymbol{b})$. To find the maximum, we employed the adaptive differential evolution optimizer, a global optimization algorithm, from the *BlackBoxOptim.jl* package (Feldt 2018).

We performed two parameter estimation and validation cases: one where all the parameters in Table 3 are shared between the two stand treatments, i.e., same parameter values were used for both treatments, with the exception of ${N}_u$ and ${k}_{\mathrm{rc},\max }$. Hydraulic conductance per leaf area (${k}_{\mathrm{rc},\max }$) is known to vary significantly among sites with water and N availability, and our hypothesis is that the carbon cost of N uptake (${N}_u$) differs between the treatments. In the second case, none of the parameters in Table 3 were shared between the two stand treatments, i.e. the parameters were estimated separately for the control and fertilized stand. The result of the first case will be shown in the Results section and the result of the second case can be viewed in Methods S4 and Figures S3–S5 available as Supplementary data at *Tree Physiology* Online*.*

**Table 3:** Estimated parameters. All parameters were shared among the stand treatments (fertilized F and control C), except when indicated by an asterisk (^*^).

Parameter	Description (units)	Estimates	
		F	C
Nitrogen cost parameter			
${N}_u$	Cost parameter of C investment to N uptake to maintain ${J}_{\mathrm{max}}$ (−)	0.0^*^	0.012^*^
GPP model parameters			
${\alpha}_{\mathrm{season}}$	Seasonal apex value of quantum yield peak (−)	0.19	0.19
${a}_{J\max }$	Ratio between ${J}_{\max, \mathrm{opt}}$ and N concentration per leaf area (${N}_{m,f}$) (mol m^−2^ s^−1^ kg leaf kg^−1^ leaf N).	0.02	0.02
$\Delta S$	Parameter controlling when the photosynthesis is at full capacity (°C)	18.29	18.29
$\tau$	Parameter controlling the temperature delay (days)	14.87	14.87
Hydraulics model parameters			
${k}_{\mathrm{sc},\max }$	Maximum soil-canopy hydraulic conductance (per leaf area) (mol m^−2^ leaf s^−1^ MPa^−1^)	0.00057^*^	0.00067^*^
Variance parameters			
${a}_{\mathrm{GPP},j}/{b}_{\mathrm{GPP},j}$	The parameters which control the variance of the error distribution for GPP (g C m^−2^ ground day^−1^/−)	0.41/0.08	0.41/0.08

### Model testing and validation

To validate our model, we excluded 20% of the datapoints (119 out of 588 datapoints) to form a validation dataset. The validation dataset was generated by randomly selecting datapoints from the complete dataset. The remaining datapoints, the training dataset, were used for parameter estimation. To minimize the effect of selection bias, we repeated the parameter estimation and validation process 10 times for both cases, thus creating 10 random validations and training datasets.

Because the representation of N limitation is a unique aspect of our model, we evaluated the impacts of excluding variation in key N variables, leaf N (${N}_{m,f}$) and N uptake costs (${N}_u$), on model results and performance in terms of the R^2^ values of GPP and *E_c_*. Three alternative simulations were performed by (i) enforcing constant leaf N concentrations (${N}_{m,f}$) over time, (ii) applying the same ${N}_{m,f}$ for both fertilization treatments and (iii) applying the same soil N uptake cost (${N}_u$) for both treatments. The results were compared to the default model case. We also evaluated the effect of removing soil water variation ($\theta$), which is another key driving variable. We tested to what degree the variability of $\theta$ effects the model predictions by running the model with the parameters from Table 2 and Table 3 with static $\theta$ values and comparing to the default case. We choose the static value of $\theta$ as the mean of the default $\theta$ time series for both stand treatments.

## Results

### The model can predict seasonal changes in $\mathrm{GPP}$ and ${E}_C$

**Figure 3:**
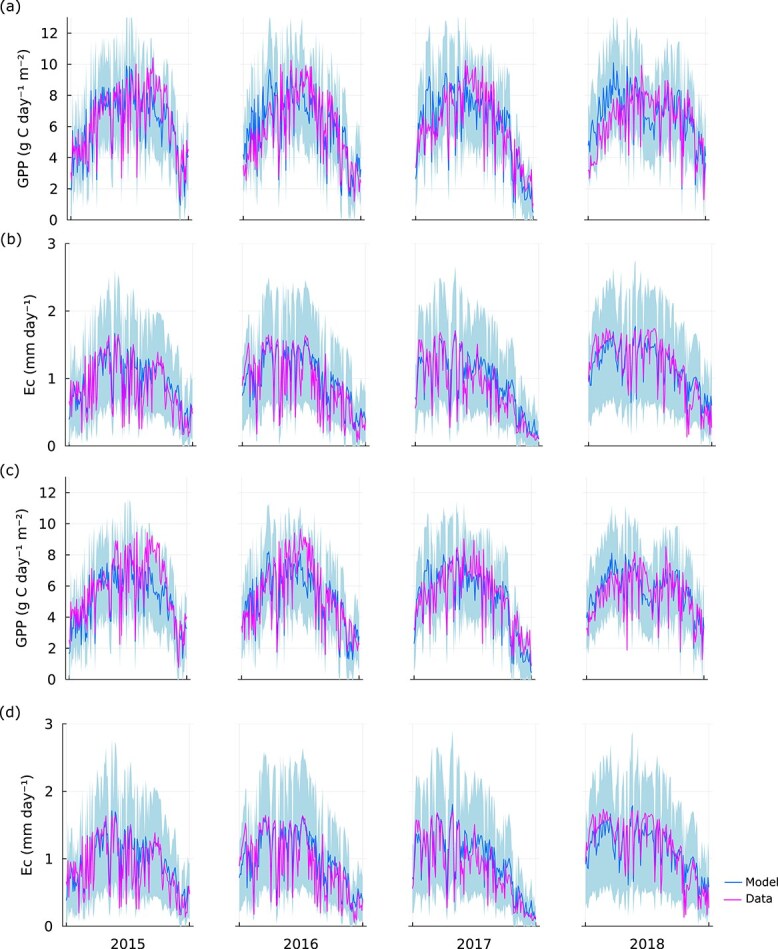
Result from the parameter estimation case when most of the parameters are shared between the two stands, fertilized (a,b) and control (c,d). Panels (a, c) and (b, d) depict the data against the corresponding simulated values for ecosystem $\mathrm{GPP}$ and canopy transpiration (${E}_C$), respectively. The 95% confidence interval of the fitted Laplace distribution (see Parameter estimation section) is depicted by the shaded area.

The result for the parameter estimation is depicted in Figure 3. Estimated parameters are provided in Table 3. The *R*^2^ in the training set for the run with the highest likelihood was 0.71 ($\mathrm{GPP}$) and 0.8 (${E}_C$) for the fertilized stand. The corresponding values for the control were 0.7 ($\mathrm{GPP}$) and 0.79 (${E}_C$) (Table 4). (Error estimates for all 10 runs are depicted in Figure S1 available as Supplementary data at *Tree Physiology* Online). Overall, our model was able to capture the inter-seasonal variation of $\mathrm{GPP}$ and ${E}_c$. However, there is a bias present when examining the model residuals (Figure S2 available as Supplementary data at *Tree Physiology* Online). Specifically, for the estimations of ${E}_c$ the model overpredicts for lower ${E}_c$ and underpredicts for lower values. For $\mathrm{GPP}$, the model underpredicts for higher GPP values.

**Table 4:** Model performance measure (RMSE, root-mean-square error; MAPE, mean absolute percentage error; *R*^2^, coefficient of determination) during the parameter estimation (training) and model validation (validation). Parameters where shared among both stand treatments (fertilized and control).

	$\mathbf{GPP}$		${\boldsymbol{E}}_{\boldsymbol{C}}$	
	Training	Validation	Training	Validation
Fertilized				
RMSE	1.14 g C m^−2^ ground day^−1^	1.05 g C m^−2^ ground day^−1^	0.2 mm day^−1^	0.19 mm day^−1^
MAPE	16.11%	16.3%	28.99%	26.11%
*R* ^2^	0.71	0.75	0.8	0.84
**Control**				
RMSE	1.04 g C m^−2^ ground day^−1^	0.81 g C m^−2^ ground day^−1^	0.21 mm day^−1^	0.19 mm day^−1^
MAPE	14.84%	14.57%	28.63%	26.04%
*R* ^2^	0.7	0.83	0.79	0.83

### The effect of soil N availability on $\mathrm{GPP}$ and ${E}_C$ is captured by hydraulic conductance per leaf area and the site-specific N cost

The summary statistics of the shared and non-shared parameter estimation cases showed similar predictive performance. For the non-shared parameter case, the *R*^2^ was 0.72 ($\mathrm{GPP}$) and 0.81 (${E}_C$) for the fertilized stand and 0.75 ($\mathrm{GPP}$) and 0.8 (${E}_C$) for the control stand (see Methods S4 and Table S2 available as Supplementary data at *Tree Physiology* Online). The result suggests that the difference between the two treatments can be well captured by the differences in N acquisition cost (${N}_u$) and hydraulic conductivity per leaf area (${k}_{\mathrm{sc},\max }$). ${N}_u$ (unitless) was 0.00 and 0.012 and ${k}_{\mathrm{sc},\max }$ was 0.57 and 0.67 mmol m^−2^ leaf s^−1^ MPa^−1^ for the fertilized and control treatments, respectively (Table 3).

### Environmental drivers of leaf N concentration, stomatal conductance and water-use efficiency

**Figure 4:**
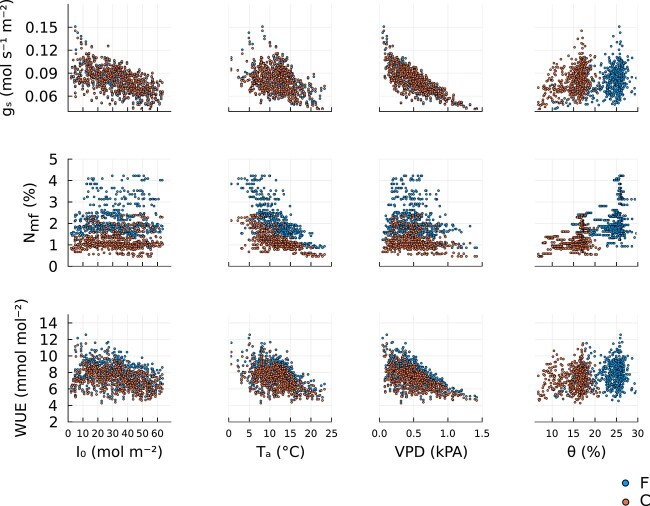
Response of the optimal, stomatal conductance (${g}_s$, first row), leaf mass-based N concentration (${N}_{m,f}$, second row) and water-use efficiency ($\mathrm{WUE}$) to change in weather variables: above canopy photosynthetic active radiance (${I}_0$, first column), mean ambient air temperature (${T}_a$, second column), vapor pressure deficit ($\mathrm{VPD}$, third column) and soil water content ($\theta$, fourth column). The blue circles correspond to the optimal trait values for the fertilized stand (F) and orange circles correspond to the control stand (C). Correlation values between traits and weather variables can be found in Table S3 available as Supplementary data at *Tree Physiology* Online. The figure was generated by applying the model to the environmental data (Figure 2) using parameters from Tables 2 and 3.

Figure 4 illustrates the variation in optimal stomatal conductance (${g}_s$) and leaf N concentration (${N}_{m,f}$) with respect to the weather variables, including irradiance (${I}_0$), mean ambient temperature (${T}_a$), vapor pressure deficit ($\mathrm{VPD}$) and soil water content ($\theta$). We calculated the Pearson correlation coefficients between the plant variables and the weather variables. For ${N}_{m,f}$ the correlation was 0.01 (*P* = 0.81), −0.7 (*P* < 0.001), −0.26 (*P* < 0.001) and 0.3 (*P* < 0.001) for ${I}_0$, ${T}_a$, $\mathrm{VPD}$ and $\theta$, respectively. For ${g}_s$ the corresponding values were −0.58 (*P* < 0.001), −0.43 (*P* < 0.001), −0.78 (*P* < 0.001) and 0.32 (*P* < 0.001). The result indicates that the optimal value of ${N}_{m,f}$ mostly respond to changes in ${T}_a$, whereas ${g}_s$ responds mostly to the change in VPD and ${I}_0$. Correlation values shown here are for the fertilized stand. The control showed similar results and these values can be viewed in Table S3 available as Supplementary data at *Tree Physiology* Online.

The response of water use efficiency, $\mathrm{WUE}=\mathrm{GPP}/{E}_c$, to meteorological variables ${I}_0$,${T}_a$, $\mathrm{VPD}$ and $\theta$ are shown in Figure 4; here, $\mathrm{GPP}$ reference to the GPP of canopy vegetation. The correlation coefficients between $\mathrm{wue}$ and meteorological variables were −0.32 (*P* < 0.001), −0.51 (*P* < 0.001), −0.61 (*P* < 0.001) and 0.16 (*P* < 0.001) for ${I}_0$, ${T}_a$, $\mathrm{VPD}$ and $\theta$, respectively. From the correlation values and Figure 4 we see that $\mathrm{wue}$ responds similarly to the various weather variables as ${g}_s$. In contrast, the $\mathrm{wue}$ response showed little resemblance to the weather response of ${N}_{m,f}$, indicating that $\mathrm{wue}$ is mainly influenced by ${g}_s$.

### The importance of N and soil water limitations for model results

The effects of variability in leaf N (${N}_{m,f}$) and N uptake costs (${N}_u$) did not exhibit strong impacts on the model’s ability to predict the observed $\mathrm{GPP}$ and ${E}_c$ variation. The use of a static ${N}_{m,f}$ value instead of a dynamically optimized and the use of equal ${N}_{m,f}$ or ${N}_u$ in both treatments, all had minimal impacts on the model’s fit to $\mathrm{GPP}$ and ${E}_c$ observations (Tables S4 and S6 available as Supplementary data at *Tree Physiology* Online). However, applying the same ${N}_u$in both treatments (${N}_u=0.006$) shifted the modeled mean ${N}_{m,f}$values to 1.5% in both treatments, which diverges strongly from the observed values (1.94% for the fertilized stand and 1.16% for the control, Lim et al. 2015). The use of static soil water content ($\theta$) had negligible impact on the model’s ability to predict observed $\mathrm{GPP}$ and ${E}_c$ (Table S5 available as Supplementary data at *Tree Physiology* Online).

## Discussion

### Model scope and limitations

Our model estimates canopy transpiration (${E}_c$) and $\mathrm{GPP}$ based on optimal acclimation of stomatal conductance and leaf N concentration. The model can, for the most part, accurately predict observed inter- and intra-seasonal variation of ${E}_c$ and $\mathrm{GPP}$ at the two study sites (Figure 3), although there is a moderate bias in the estimations of ${E}_c$ and underprediction of $\mathrm{GPP}$ for higher $\mathrm{GPP}$ data values (Figure S2 available as Supplementary data at *Tree Physiology* Online). An exception is the growing season of 2018, where the site was hit by a severe drought period with very high $\mathrm{VPD}$ and low soil water in the middle of the growing season (Figure 2). During 2 weeks in this period the model somewhat underestimates $\mathrm{GPP}$ and ${E}_c$ for both the fertilized stand and the control, indicating an exaggerated reduction of stomatal conductance. This divergence may be caused by an underestimation of the actual soil water content available to the trees since soil water was measured at a maximum depth of 50 cm and the trees may have deeper roots. Rooting depth of these trees has not been measured, but Scots pine often has tap roots extending much deeper than 50 cm (Martinsson 1986).

Our results demonstrate that the effect of fertilization can be captured by adjustments in only two parameters: maximum soil-to-canopy hydraulic conductance per leaf area, ${k}_{\mathrm{sc},\max }$, and the site-specific N acquisition cost parameter, ${N}_u$. The current version of the model predicts $\mathrm{GPP}$ and transpiration for trees with a given $\mathrm{LAI}$ and potential hydraulic conductivity per $\mathrm{LAI}$ (${k}_{\mathrm{sc},\max }$). As such, it is naturally unable to predict dynamics of these properties, which could be addressed in future versions of the model (see Outlook below).

### The impact of increased soil N availability

The difference between our model and the previous models by Eller et al. (2018) and Prentice et al. (2014) is that we do not only account for optimal stomatal response but also optimize leaf N concentration. Another recent model that allows optimization of stomatal conductance and ${J}_{\mathrm{max}}$, which is functionally equivalent to our optimization of leaf N, is the model by Joshi et al. (2022). However, this model has a different hydraulic cost function and, more importantly, does not address variation in the cost of N uptake related to soil N availability.

Our model allows us to account for the response to increased soil N availability. In agreement with our model predictions, it has been observed that increased soil N availability results in increased leaf N concentration (Lim et al. 2015, Tarvainen et al. 2016). This, in turn, has a positive impact on the potential photosynthetic capacity and the ${J}_{\mathrm{max}}$ in our model. The model results suggest that the fertilization treatment radically reduced the trees’ C cost for N uptake (the unitless cost parameter ${N}_u$) from 0.012 to 0.0. However, without further empirical evidence, the zero cost of N uptake should be interpreted with care, rather as being too low to be separated from other costs by the model analysis than as an absolute zero value. Nevertheless, the large difference in N uptake costs agrees with the concurrent observed difference in C allocation to the components contributing to N uptake: fine-root, mycorrhiza and exudates production (Marshall et al. 2023). The low-cost uptake of N may be possible via mass flow of dissolved N in water uptake, which can be the dominant process of N uptake in fertilized conditions (Oyewole et al. 2017, McMurtrie and Näsholm 2018, Henriksson et al. 2021) where the chemical profile of soil N is biased toward mineral forms (ammonium and nitrate, Inselsbacher et al. 2014) that are mobile in the soil solution. This effect may have been further enhanced by higher soil water in the fertilized stand related to a higher field capacity and more organic matter than in the control stand (Tian et al. 2021). Other than fertilized soils, this may occur under specific conditions where soil nitrification rates are high. In contrast, the control stand represents a more common boreal forest soil profile, where N is present mainly in organic and less mobile forms, which drives up the N acquisition cost.

Besides the decrease in ${N}_u$, we also get an increase in $\mathrm{LAI}$ in the fertilized stand. Because the sapwood cross sectional area has not increased to the same extent, we get a 12% lower Huber value (sapwood area/leaf area) in the fertilized compared with the control stand (calculated from field measurements). This contributes to the estimated 15% decrease in estimated conductivity per leaf area (${k}_{sc,\max }$) in the fertilized stand. These results may reflect an optimality response to a lower cost of N uptake and thus lower cost of leaf area.

Given the novel representation of N limitation in our model, a relevant question is how important is N limitation for accurate $\mathrm{GPP}$ modeling? Based on the negligible impacts on the model’s ability to reproduce measured $\mathrm{GPP}$ and ${E}_c$ of removing temporal- and treatment differences in leaf N (Table S4 available as Supplementary data at *Tree Physiology* Online) it may appear unimportant. The same result is also found for temporal variation in soil water content $\theta$ (Table S5 available as Supplementary data at *Tree Physiology* Online), which is not too surprising since Figure 4 showed a low correlation between $\theta$ and ${g}_s$ as well as $\theta$ and ${N}_{m,f}$.

However, these results are not necessarily evidence of irrelevance of the N-related variables but rather a lack of observational constraints. As the model is calibrated to reproduce observed GPP and ${E}_c$ only, it can compensate for lacking effects of N or soil water by adjustments of parameters, even though they may have side effects on other processes and variables that are not constrained by observations. This is obvious from the results of applying the same N uptake cost in both treatments—it does not deteriorate the GPP and ${E}_c$ predictions (Table S6 available as Supplementary data at *Tree Physiology* Online), but it leads to unrealistic leaf N concentrations. Thus, additional data, such as observations of seasonal variation in leaf N, would be valuable for better quantification of the importance of N limitation in our GPP modeling approach.

### The responses of leaf variables and water-use efficiency to weather variables

Based on our results, we can infer that the optimal leaf N concentration (${N}_{m,f}$) is negatively correlated with temperature (Figure 4). This is in line with empirical observations for Scots pine (Zha et al. 2002), understory evergreen plants (Muller et al. 2011) and a global biogeographic pattern of different plant species (Reich and Oleksyn 2004). This is caused by the shape of the C assimilation curve and the cost of maintaining ${J}_{\mathrm{max}}$ as a function of ${N}_{m,f}$. The optimum ${N}_{m,f}$ will occur when the slope of the C assimilation function is equal to the slope of the cost line. When the temperature increases, the slope of the cost line increases more than the C assimilation curve, and thus, the optimal ${N}_{m,f}$ will decrease (Figure S8 available as Supplementary data at *Tree Physiology* Online). This temperature effect further implies that the leaf N content should decrease with higher temperatures in the middle of the growth season (see Figure S7 available as Supplementary data at *Tree Physiology* Online). Because the model only accounts for photosynthetic N but no other forms (e.g. N for structural purposes or storage) that may respond differently, it probably overestimates the seasonal N variation. Furthermore, the model does not consider seasonal dynamics in the vertical N distribution in the canopy. Nevertheless, qualitatively similar seasonal variation has been observed in a nearby boreal Scots pine forest (Näsholm and Ericsson 1990) as well as a temperate Scots pine forest (Wyka et al. 2016).

As expected, we found that stomatal ${g}_s$ correlated best with $\mathrm{VPD}$. A high $\mathrm{VPD}$ leads to an increase in water loss and negative water potential for a fixed value of ${g}_s$. Thus, ${g}_s$ is reduced in response to rising $\mathrm{VPD}$, in order to reduce the hydraulic risk cost. Our results (Figure 4) further show that the stomatal ${g}_s$ is negatively correlated to the above canopy photosynthetic active radiance (${I}_0$). However, this correlation does not correspond to a direct relationship but reflects a correlation between $\mathrm{VPD}$ and ${I}_0$ and confounding variation in other drivers, which was confirmed by running the model with fixed weather variables (except for ${I}_0$) (Figure S9 available as Supplementary data at *Tree Physiology* Online). Furthermore, we found that the response of $\mathrm{WUE}$ to the environmental variables closely follows that of ${g}_s$ (Figure 4). Thus, $\mathrm{wue}$ is more strongly correlated to ${g}_s$ than ${N}_{m,f}$, which is not surprising as water used as transpiration is directly regulated by ${g}_s$. In all cases, soil water content ($\theta$) had a small impact on ${g}_s$, ${N}_{m,f}$ and $\mathrm{WUE}$ when $\theta$ is large (~$\theta >25\%$ for the fertilized stand and $\theta >15\%$ for the control stand). For lower soil water content, a decrease in $\theta$ leads to decreases in ${g}_{s,\mathrm{top}}$, ${N}_{m,f,\mathrm{top}}$ and $\mathrm{wue}$, driving the observed positive correlation between $\theta$ and the plant variables and process rates. The difference between high and low $\theta$ is caused by the monotonically decreasing concave down shape of the vulnerability function, $P\left(\psi \right)$. For low values of $\theta$ (highly negative soil water potential, ${\psi}_s$), the increase in cost associated with small changes in plant variables (the derivative of the cost with respect to the plant variables) is larger than for higher $\theta$.

In summary, while both ${g}_s$ and ${N}_{m,f}$ correlated well with different weather variables, there is no significant correlation between the two plant variables (Pearson correlation = 0.014, *P* = 0.73 for the fertilized stand and 0.00072, *P* = 0.99 for the control stand, Figure S6 available as Supplementary data at *Tree Physiology* Online). This implies that ${g}_s$ and ${N}_{m,f}$ mostly respond to different meteorological variables. ${g}_s$ responds strongly to irradiance and $\mathrm{VPD}$, while ${N}_{m,f}$ responds strongly to the ambient temperature.

### Outlook

The key scientific advancement made by this improved model lies in its ability to explain, and accurately predict the interacting effects of climate, soil water availability and soil N availability on $\mathrm{GPP}$ and transpiration, based on an eco-evolutionary optimality principle (EEO), i.e. optimization based on eco-evolutionary theory. This capacity makes the model well-suited for studying the impact of climate change in N-limited boreal forests. Also, the effects of changes in soil N availability, such as fertilization practices and N deposition, can be studied by our model. However, one has to be careful when applying the model to severe drought conditions as it does not yet account for accumulation of hydraulic damages (Franklin et al. 2023). A challenge in applying our proposed model to other stands is the estimation of the carbon cost of N uptake, ${N}_u$. In future studies, this parameter could be estimated for different experimental stands and statistically linked to soil conditions at the stands, such as a nutrient limitation index (Van Sundert et al. 2020). The resulting relationships can then be used to model ${N}_u$ at other sites. To address long-term effects on forest growth, the model can be extended with the capacity to predict carbon allocation to the different plant organs, such as branches, fine roots, stems and leaves. To this end, our model could be coupled with allocation models based on similar EEO principles (e.g. Franklin et al. 2012, Fransson et al. 2021).

## Supplementary Material

An_eco-physiological_model_of_forest_photosynthesis_and_transpiration_SD_tpae168

## Data Availability

The code for the model is available on GitHub at https://github.com/PeterFransson/CCPH.jl, while the scripts and data used for fitting the model and running simulations can be found at https://github.com/PeterFransson/CCPH_Project.
